# Preparation of La_2_(WO_4_)_3_/CuWO_4_ composite nanomaterials with enhanced sonodynamic anti-glioma activity

**DOI:** 10.3389/fbioe.2025.1566946

**Published:** 2025-03-20

**Authors:** Fang-Yu Liu, Xin Wang, Ye-Fu Liu

**Affiliations:** ^1^ The First Clinical College, Liaoning University of Traditional Chinese Medicine, Shenyang, China; ^2^ Shenyang Key Laboratory for Causes and Drug Discovery of Chronic Diesases, Liaoning University, Shenyang, China; ^3^ Liaoning Cancer Hospital and Institute, Shenyang, China

**Keywords:** La_2_(WO_4_)_3_/CuWO_4_ composite, nano-sonosensitizer, sonodynamic therapy (SDT), sonodynamic antitumor activity, reactive oxygen species (ROS)

## Abstract

**Introduction:**

Sonodynamic therapy (SDT) is an innovative way to treat tumors by activating sonosensitizers via ultrasound (US). The development of sonosensitizers with high sonodynamic activity is the key to promote the clinical application of SDT.

**Methods:**

In this study, a novel sonosensitizer, La_2_(WO_4_)_3_/CuWO_4_ composite LC-10, was prepared by two-step hydrothermal method and characterized. In addition, the sonodynamic antitumor activity of La_2_(WO_4_)_3_/CuWO_4_ composite LC-10 was investigated using u251 glioma cells as a model.

**Results and Discussion:**

The results showed that compared with La_2_(WO_4_)_3_ and CuWO_4_, La_2_(WO_4_)_3_/CuWO_4_ composite had better sonodynamic antitumor activity, and LC-10 had good biosafety at concentrations below 50 μg/mL. After La_2_(WO_4_)_3_ and CuWO_4_ formed La_2_(WO_4_)_3_/CuWO_4_ composite, the recombination of electron-hole (*e* ^−^–*h* ^+^) pairs were effectively inhibited, and more strongly oxidizing ROS was produced, inducing apoptosis of u251 glioma cells. In which, singlet oxygen (^1^O_2_) and hydroxyl radical (·OH), especially the production of ⋅OH, played an important role in the La_2_(WO_4_)_3_/CuWO_4_ composite mediated SDT antitumor process. The results of this study would offer a foundation for the design of CuWO_4_ base nano-sonosensitizers and its further clinical application in SDT antitumor. In addition, it also provided a new strategy for the design and development of novel nano-sonosensitizers with excellent sonodynamic activity.

## 1 Introduction

Sonodynamic therapy (SDT) is an emerging non-invasive cancer treatment derived from photodynamic therapy (PDT) (Canavese et al., 2018). SDT uses the focusing of ultrasound (US) and strong penetration of biological tissues to enrich tumor sites of sonosensitizers with certain frequency and intensity of US, and activates the sonosensitizers to produce cytotoxicity, thus producing antitumor effects (Rosenthal et al., 2004). In 1993, Umemura et al. (Umemura et al., 1993) named this method of combining sonosensitizer with US for tumor treatment as SDT according to the name of PDT. This method not only gets rid of the disadvantage of PDT’s poor tissue permeability (Hachimine et al., 2007a), but also shows unique advantages in the non-invasive treatment of deep tumors due to its local cytotoxic effect, which can minimize the damage of normal tissues around the tumor site (Yang et al., 2021). Therefore, SDT has attracted wide attention since it was proposed (Han et al., 2018; Wu et al., 2022).

According to the action mechanisms of SDT, the performance of sonosensitizers plays a crucial role in the SDT process. The traditional organic sonosensitizers used in early studies mainly include hematoporphyrin (Hp) (Umemura et al., 1989) and its derivatives such as ATX-70 (Sasaki et al., 1998), DCPH-P-Na(I) (Hachimine et al., 2007b), haematoporphyrin monomethyl ether (HMME) (Li et al., 2008) and protoporphyrin IX (PPIX) (Liu et al., 2006). In addition, some antitumor drugs and small molecule drugs such as curcumin (Jiang et al., 2020), acridine orange (Xing et al., 2021), methylene blue (He et al., 2015a), promethazine hydrochloride (He et al., 2011a), dioxypromethazine hydrochloride (He et al., 2011b), eosin B (He et al., 2015b), brilliant cresyl blue (Wang et al., 2018), toluidine blue and azure A (Qian et al., 2024) have also been proved to have good sonodynamic activity. The advantages of these organic sonosensitizers are that they all have clear chemical structures and excellent biodegradation rates. However, most of them have disadvantages such as greater hydrophobicity and phototoxicity, as well as lower tissue selectivity and stability when applied *in vivo* (Wu et al., 2022; Xing et al., 2021).

In recent decades, the research of inorganic nanoparticle based sonosensitizers such as titanium dioxide (TiO_2_) has made great progress (Yang et al., 2021). Compared with organic small molecule drugs, inorganic nanomaterials have the advantages of excellent physical and chemical properties, easy manufacturing, low phototoxicity, good biocompatibility and stability (Yang et al., 2021; Li et al., 2023). At the same time, various inorganic nanomaterials such as TiH_1.924_ (Gong et al., 2020), BaTiO_3_ (Zhu et al., 2020) and Bi_2_MoO_6_ (Dong et al., 2021) were proved to have good sonodynamic activity. These inorganic nano-sonosensitizers absorb the energy generated by the US cavitation effect (thermal and sonoluminescence) and excite the sonosensitizers to produce electron-hole (*e* ^−^–*h* ^+^) separation. The separated *e* ^−^ and *h* ^+^ migrate to the surface of the sonosensitizer to produce corresponding reduction and oxidation reactions, resulting in a large number of reactive oxygen species (ROS) generation and antitumor effects (Li et al., 2023). However, due to the rapid recombination of *e* ^−^ and *h* ^+^ in the band structure, the efficiency of ROS generation of single-component sonosensitizer is relatively low, which affects the effect of SDT (Li et al., 2023; Ping et al., 2023). Therefore, the researchers overcome the recombination of carriers by forming oxygen defect layers on the surface of sonosensitizer (Zhou et al., 2022; Wang Y. et al., 2023), deposition of precious metals (Zhang et al., 2021), ion doping (Cheng et al., 2024), construction of heterojunction (Chen et al., 2022; Kang et al., 2022) and a combination of these strategies (Zhang et al., 2023a; Song et al., 2022), thus further improving the efficiency of SDT.

Because of the effective conversion of light energy into thermal and chemical energy, tungstate nanomaterials are often used as photoresponsive materials in the field of photocatalytic degradation of organic pollutants (Kumar et al., 2022), photothermal therapy (Xiao et al., 2016), PDT and radiotherapy (Zhang et al., 2018). CuWO_4_ is an important semiconductor material with a band gap of about 2.60 eV (Liu et al., 2023). Compared with other tungstate structures, CuWO_4_ exhibits stronger absorption in the near infrared region, indicating that CuWO_4_ is a good candidate for PDT. For example, Cui et al. (2021) prepared a single original nanostructured CuWO_4_ nanodots, and introduced the nanodots into tumor tissue to generate ROS to generate PDT under 808 nm light irradiation, and released copper ions into the acidic tumor microenvironment to promote Fenton-like reaction and generate chemodynamic therapy, which can effectively inhibit tumor tissue growth. Since most sonosensitizers are derived from photosensitizers, CuWO_4_ nanomaterials may be a candidate sonosensitizer for SDT applications.

The construction of heterojunction can effectively inhibit the *e* ^−^–*h* ^+^ pairs recombination, promote the generation of ROS, and significantly improve the effect of SDT (Ping et al., 2023; Chen et al., 2022; Kang et al., 2022). Therefore, in this paper, La_2_(WO_4_)_3_/CuWO_4_ composites were prepared by two-step hydrothermal method, and the microstructure, morphology and elemental composition of La_2_(WO_4_)_3_/CuWO_4_ composites were analyzed by X-ray diffraction (XRD), scanning electron microscope (SEM), energy dispersive X-ray spectroscopy (EDX) and X-ray photoelectron spectroscopy (XPS). Using u251 glioma cells as model, the sonodynamic anti-glioma activity of La_2_(WO_4_)_3_/CuWO_4_ composites was investigated by MTT method. Finally, the sonodynamic antitumor mechanism of La_2_(WO_4_)_3_/CuWO_4_ composites was discussed based on the optical properties and electrochemical characteristics of La_2_(WO_4_)_3_/CuWO_4_ composites and ROS probe experimental results. It is expected that the results of this study will provide the research basis for the further development and application of CuWO_4_ based sonosensitizers in SDT antitumor.

## 2 Experimental sections

### 2.1 Materials and reagents

NaWO_4_·2H_2_O (AR), 1, 3-diphenylisobenzofuran (DPBF, AR) and terephthalic acid (TA, AR) were purchased from Tianjin Hengxing Chemical reagent manufacturing Co., LTD., (China). NaOH (AR) was purchased from Tianjin Yongda Chemical reagent Co., LTD., (China). Anhydrous ethanol (AR), Cu(NO_3_)_2_·3H_2_O and La(NO_3_)_3_·6H_2_O were purchased from Tianjin Damao chemical reagent factory (China). Phosphate buffer (PBS, BR), thiazole blue (AR), RPMI Medium 1640 (BR), superior fetal bovine serum (BR), 0.25% trypsin/EDTA digestion solution (BR), serum-free cryopreservation (BR), dimethyl sulfoxide (DMSO, BR), 3-(4,5-dimethylthiazol-2-yl)-2,5 diphenyl-tetrazolium bromide (MTT, BR) and AO/EB Kit (LR) were purchased from Beijing Solarbio Technology Co., LTD., (China). Human glioma u251 cells were purchased from Cell Resource Center, Institute of Basic Medicine, Chinese Academy of Medical Sciences.

### 2.2 Preparation of La_2_(WO_4_)_3_/CuWO_4_ composite

1.0872 g Cu(NO_3_)_2_·3H_2_O and 0.2165 g La(NO_3_)_3_·6H_2_O were dissolved together in 40 mL deionized water. 2.3090 g Na_2_WO_4_·_2_H_2_O was weight and dissolved in 20 mL deionized water. Then, under the condition of magnetic stirring, the Na_2_WO_4_ solution was added to the mixed solution of Cu(NO_3_)_2_ and La(NO_3_)_3_ drop by drop, and continued to stir for 30 min. Then the mixed solution was transferred to the polytetrafluoroethylene inner tank of 100 mL stainless steel reactor and reacted in a constant temperature drying oven at 180°C for 24 h. The reaction products were washed with deionized water and anhydrous ethanol three times respectively, and dried at 60°C for 8 h to obtain La_2_(WO4)_3_/CuWO_4_ composite (LC-10) sample. According to the above experimental methods, CuWO_4_ sample was prepared without adding La(NO_3_)_3_·6H_2_O in the process of synthesis. Similarly, La_2_(WO_4_)_3_ sample was prepared under the above experimental conditions without adding Cu(NO_3_)_2_·3H_2_O in the process of synthesis. Detailed information on the various instruments used in the paper could be found in Supplementary Material.

### 2.3 Cytotoxicity assay

The cytotoxicity of nano-sonosensitizers and nano-sonosensitizers-mediated sonodynamic action on u251 glioma cells was tested by the typical MTT assay. The u251 glioma cells were plated in 96-well microplates (6 × 10^3^ cells per well) and incubated at 37°C for 24 h. After removing the culture medium, 50 μL of base medium containing various concentrations of La_2_(WO_4_)_3_, CuWO_4_ and LC-10 samples (10, 20, 50, 100 and 200 μg/mL) were added. After incubation at 37°C for 24 h, 20 μL of medium containing MTT (0.5 mg/mL diluted with medium) was added to each well and continued incubation at 37°C for 4 h. Then, the supernatant in the well was discarded, 150 μL of DMSO was added to each well, and the optical density (OD) was measured at 490 nm using a microplate reader instrument. The cell viability was calculated by Graphpad Pism software. In the experiment on sonodynamic damage to u251 glioma cells, various concentrations of La_2_(WO_4_)_3_, CuWO_4_ and LC-10 samples (0, 10, 20, 30, 40 and 50 μg/mL) were added. After incubation at 37°C for 24 h, the cells were irradiated with a 1.0 MHz US probe for 1 min, and incubated at 37°C for 24 h. MTT assay was used to detect cell viability.

### 2.4 Detection of ROS

In order to detect the formation of singlet oxygen (^1^O_2_) in the SDT process, DPBF was used as a probe, which could react with ^1^O_2_ to decompose DPBF into 1, 2-diphenyl-benzene, resulting in a decrease in the absorption intensity of its characteristic absorption peak at 410 nm (Zhou et al., 2024; Zhang et al., 2023b). The specific experimental method was as follows: 8 mL of 75% ethanol solution containing DPBF (8 mg/L) and sonosensitizer (20 μg/mL) were taken and placed in the US device for 5 min. Then centrifuged at 15,000 rpm for 10 min to remove the sonosensitizer. The absorption spectra of the solution near 410 nm were measured with an ultraviolet-visible (UV-Vis) spectrophotometer.

In order to detect the formation of hydroxyl radical (⋅OH) in the SDT process, TA was used as a probe, which could react with ⋅OH to produce 2-hydroxyterephthalic acid, resulting in a characteristic fluorescence emission peak generated around 430 nm (Pan et al., 2018). Typically, 8 mL 0.5 mmol/L TA solution containing 20 μg/mL of sonosensitizer was taken, then the solution was placed in US bath for US irradiation for 5 min, and centrifugated at 15,000 rpm for 10 min to remove the sonosensitizer. The emission spectrum of the supernatant near 425 nm was measured by a fluorescence spectrophotometer with excitation wavelength of 315 nm.

### 2.5 Statistical analysis

All data were expressed as mean ± SD, and one-way ANOVA was performed by Graphpad Pism software. When *P* was less than 0.05, it was statistically significant.

## 3 Results and discussions

### 3.1 Characterization of La_2_(WO_4_)_3_/CuWO_4_ composite

**FIGURE 1:**
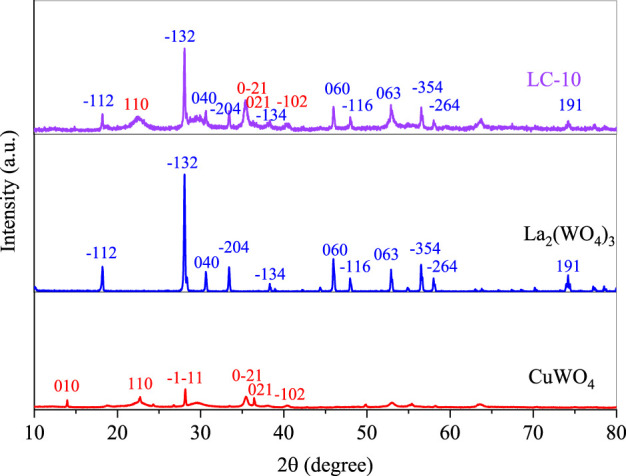
XRD patterns of the prepared La_2_(WO_4_)_3_, CuWO_4_ and LC-10 composite.

The XRD patterns of the prepared La_2_(WO_4_)_3_, CuWO_4_ and LC-10 composite were shown in Figure 1. As shown in Figure 1, the XRD patterns of CuWO_4_ showed that the diffraction peaks were at 13.96°, 22.73°, 28.17°, 35.46°, 36.43° and 40.52°, which belonged to the (010), (110), (−1–11), (0–21), (021) and (−102) planes of CuWO_4_, respectively. For La_2_(WO_4_)_3_, the diffraction peaks at 18.20°, 28.08°, 30.64°, 33.44°, 38.34°, 45.98°, 47.98°, 52.92°, 56.52°, 58.02° and 74.20° were related to the planes (−112), (−132), (040), (−204), (−134), (060), (−116), (063), (−354), (−264) and (191) of La_2_(WO_4_)_3_. For LC-10 composite, a series of characteristic peaks of CuWO_4_ and La_2_(WO_4_)_3_ appeared. The XRD diffraction peaks at 22.52°, 35.40°, 36.32° and 40.50° were attributed to the (110), (0-21), (021) and (−102) crystal planes of CuWO_4_. The XRD diffraction peaks at 18.22°, 28.10°, 30.68°, 33.44°, 38.32°, 45.98°, 47.98°, 52.90°, 56.54°, 58.02° and 74.20° were attributed to the (−112), (−132), (040), (−204), (−134), (060), (−116), (063), (−354), (−264) and (191) crystal planes of La_2_(WO_4_)_3_. The above results confirmed the successful preparation of La_2_(WO_4_)_3_/CuWO_4_ composite.

**FIGURE 2:**
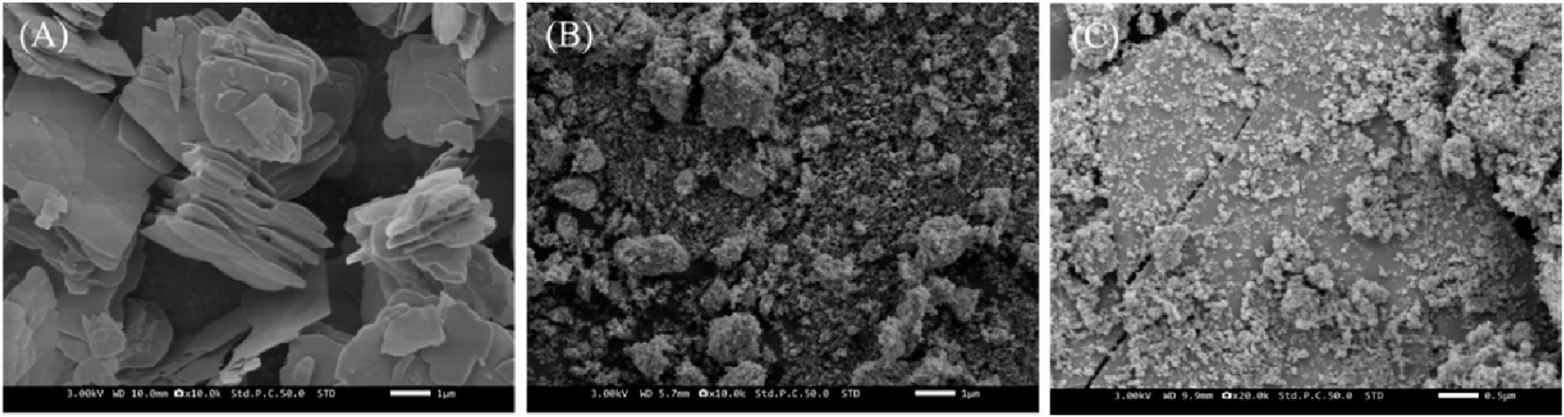
SEM images of synthesized La_2_(WO_4_)_3_ **(A)**, CuWO_4_ **(B)** and LC-10 **(C)** samples.

**FIGURE 3:**
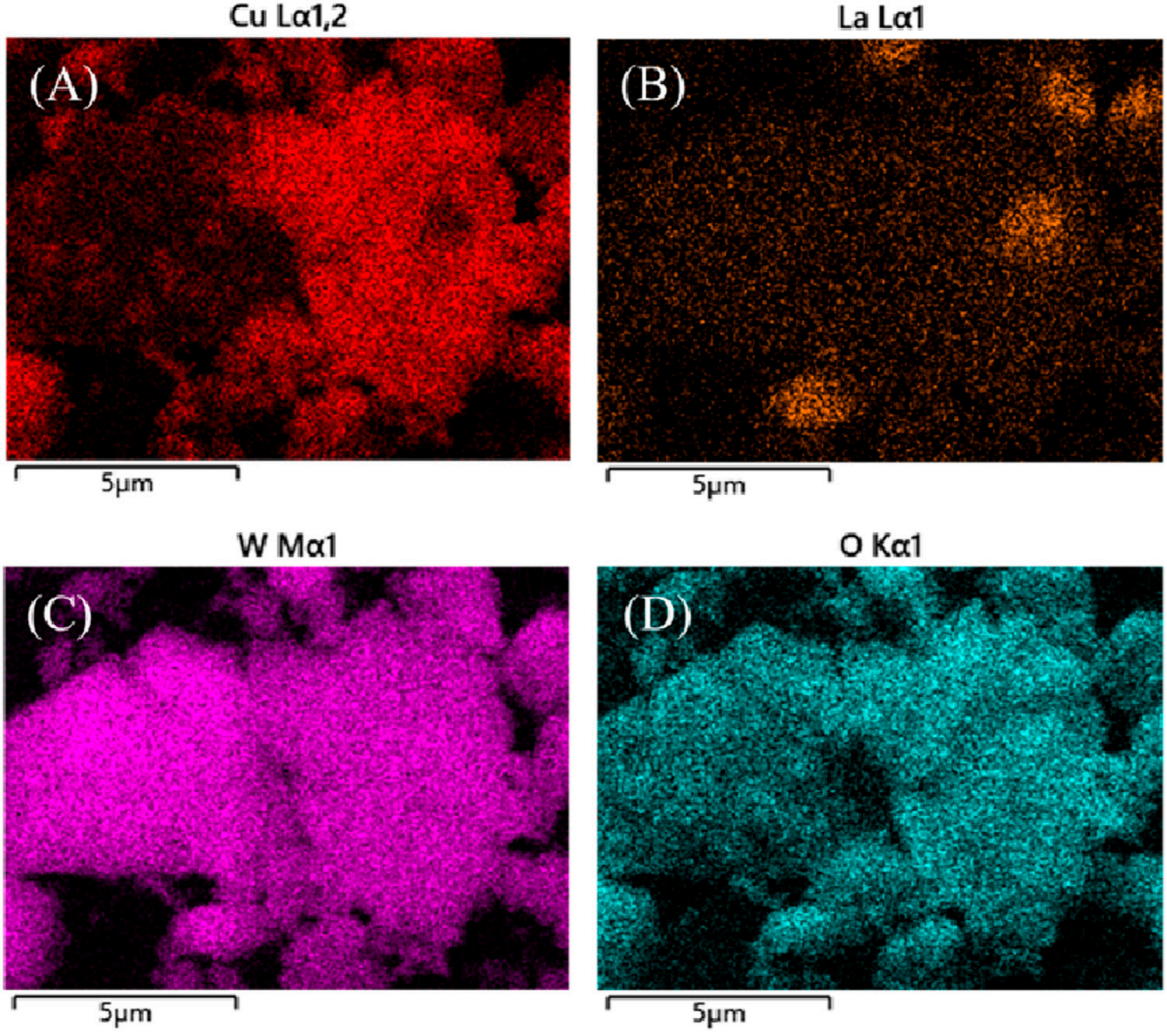
EDX diagrams of **(A)** Cu, **(B)** La, **(C)** W and **(D)** O elements in the synthesized LC-10 sample.

The morphologies of the prepared La_2_(WO_4_)_3_, CuWO_4_ and LC-10 samples were analyzed by SEM, and the results were shown in Figure 2. It could be seen that the prepared La_2_(WO_4_)_3_ sample had an irregular lamellar structure (Figure 2A), and the prepared CuWO_4_ sample had a nanoparticle shape (Figure 2B). It could be seen from the SEM image of the prepared LC-10 sample (Figure 2C) that CuWO_4_ nanoparticles were evenly distributed on the lamellar structure of La_2_(WO_4_)_3_, which proved the successful composite of La_2_(WO_4_)_3_ and CuWO_4_. The EDX diagrams of Cu, La, W and O elements in the synthesized LC-10 sample were shown in Figure 3. It could be seen that Cu, La, W and O elements were evenly distributed on the surface of the synthesized LC-10 sample.

**FIGURE 4:**
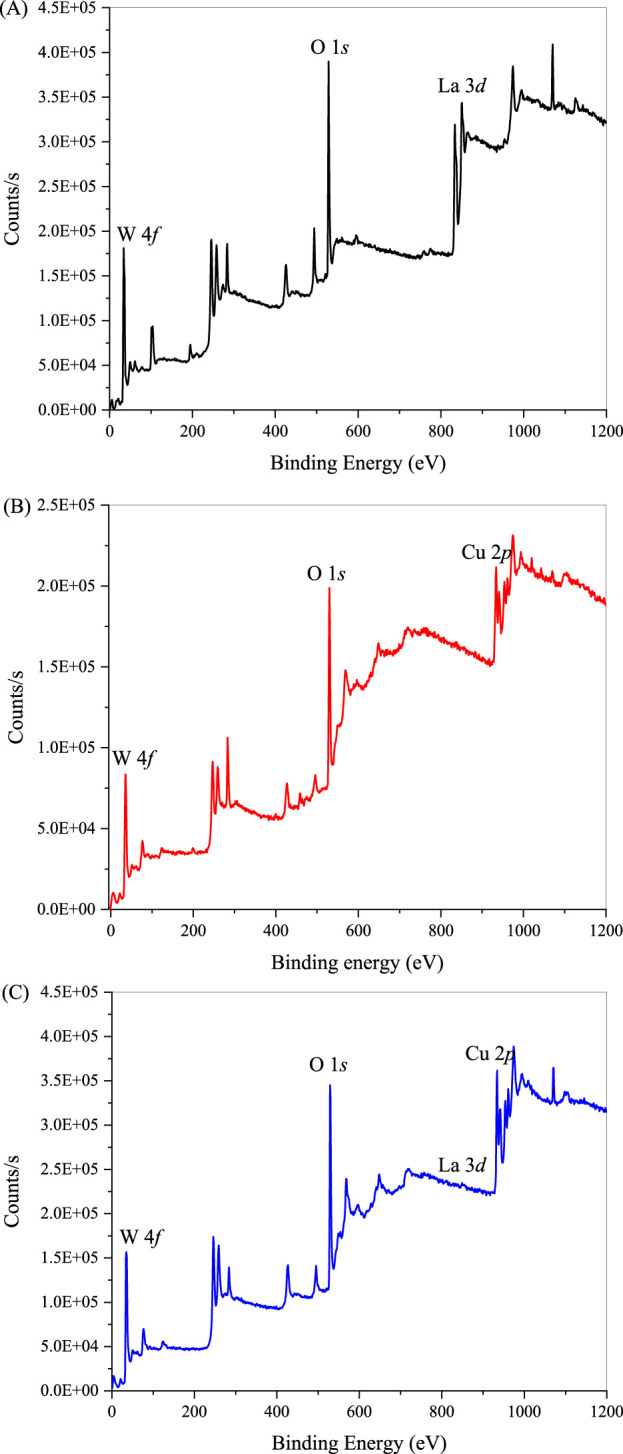
XPS spectra of synthesized La_2_(WO_4_)_3_ **(A)**, CuWO_4_ **(B)** and LC-10 **(C)** samples.

The elemental compositions and chemical valence states of synthesized La_2_(WO_4_)_3_, CuWO_4_ and LC-10 samples were analyzed by XPS measurement. As shown in Figure 4A, the three peaks of La_2_(WO_4_)_3_ sample at 832.2, 32.6 and 527.7 eV were attributed to La 3*d*, W 4*f* and O 1*s*, respectively. As shown in Figure 4B, the three peaks of the CuWO_4_ sample at 932.2, 31.1 and 528.1 eV were attributed to Cu 2*p*, W 4*f* and O 1*s*, respectively. As shown in Figure 4C, the four peaks of LC-10 composite at 833.3, 932.5, 33.7 and 528.1 eV were attributed to La 3*d*, Cu 2*p*, W 4*f* and O 1*s*, respectively.

**FIGURE 5:**
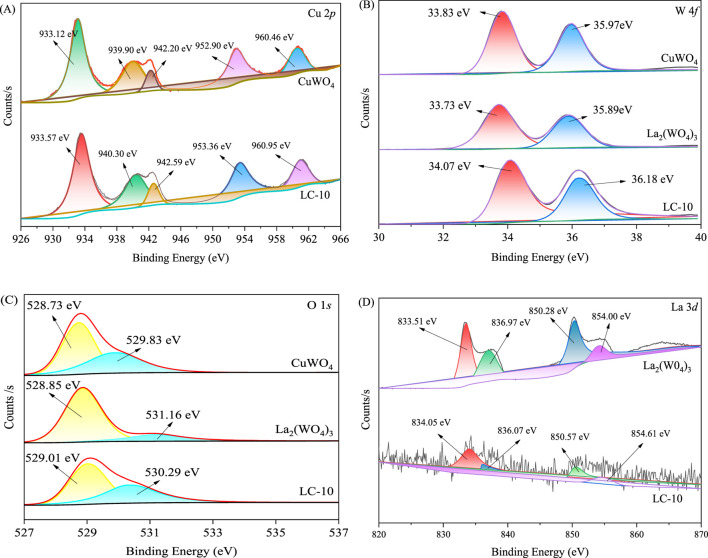
High-resolution XPS spectra of Cu 2*p* **(A)**, W 4*f* **(B)**, O 1*s* **(C)** and La 3*d* **(D)** in synthesized CuWO_4_, La_2_(WO_4_)_3_ and LC-10 samples.

The high-resolution XPS spectra of Cu 2*p*, W 4*f*, O 1*s* and La 3*d* in the synthesized CuWO_4_, La_2_(WO_4_)_3_ and LC-10 samples were shown in Figure 5. The two peaks with binding energies of 933.12 and 952.90 eV in the spectrum of Cu 2*p* of CuWO_4_ (Figure 5A) belonged to Cu 2*p*_3/2_ and Cu 2*p*_2/1_, respectively, which suggested that the presence of Cu^2+^ and the capture of photogenerated electrons by Cu^2+^ may cause the valence state change from Cu(II) to Cu(I) (Wang X. et al., 2023; Wan et al., 2013). Compared with CuWO_4_, the two fitted peaks of Cu 2*p* of LC-10 composite sample shifted slightly to the higher energy, located at 933.57 and 953.36 eV, respectively. In the high resolution XPS spectrum of W 4*f* of CuWO_4_ (Figure 5B), the two peaks at the binding energies of 33.83 and 35.97 eV belonged to W 4*f*_7/2_ and W 4*f*_5/2_, respectively, suggesting the existence of W^6+^ in the synthesized sample (Wang et al., 2021; Xu et al., 2023). The two fitted peaks of W 4*f* of La_2_(WO_4_)_3_ were located at 33.73 and 35.89 eV, respectively. Compared with CuWO_4_ and La_2_(WO_4_)_3_, the two fitted peaks of W 4*f* of LC-10 composite sample shifted slightly to the higher energy, located at 34.07 and 36.18 eV, respectively. The high-resolution XPS spectra of O 1*s* of CuWO_4_ shown in Figure 5C displayed two fitted peaks at 528.73 and 529.83 eV, respectively, indicating the presence of the lattice oxygen and O-H bonds absorbed on surface of the synthesized sample (He et al., 2024; Wei et al., 2023). The two fitted peaks of O 1*s* of La_2_(WO_4_)_3_ were located at 528.85 and 531.16 eV, respectively. Compared with CuWO_4_ and La_2_(WO_4_)_3_, the two fitted peaks of O 1*s* of LC-10 composite sample shifted slightly to the higher energy or lower energy, located at 529.01 and 530.29 eV, respectively. The high-resolution XPS spectrum of La 3*d* of La_2_(WO_4_)_3_ shown in Figure 5D displayed the spin–orbit splitting: La 3*d*_5/2_ (833.51 and 836.97 eV) and La 3*d*_3/2_ (850.28 and 854.00 eV), which were in accordance with the standard XPS peaks of La^3+^ (Baby et al., 2024). Compared with La_2_(WO_4_)_3_, the fitted peaks of La 3*d* of LC-10 composite sample also shifted slightly. The above results indicated that the two substances, CuWO_4_ and La_2_(WO_4_)_3_ interacted with each other, and the heterojunction of La_2_(WO_4_)_3_/CuWO_4_ formed.

**FIGURE 6:**
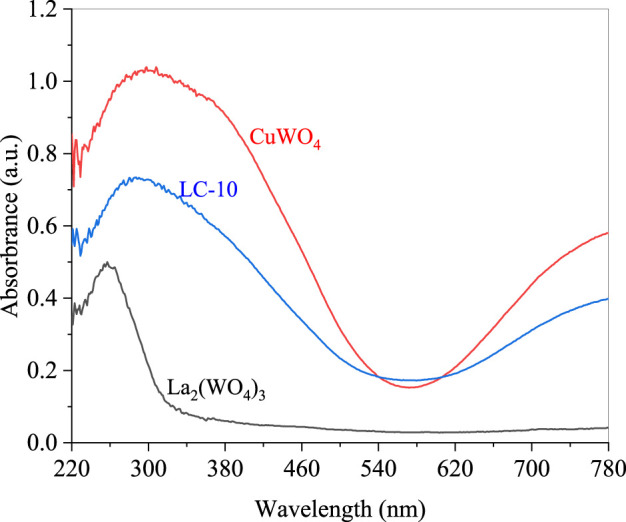
DRS spectra of CuWO_4_, La_2_(WO_4_)_3_ and LC-10 samples.

**FIGURE 7:**
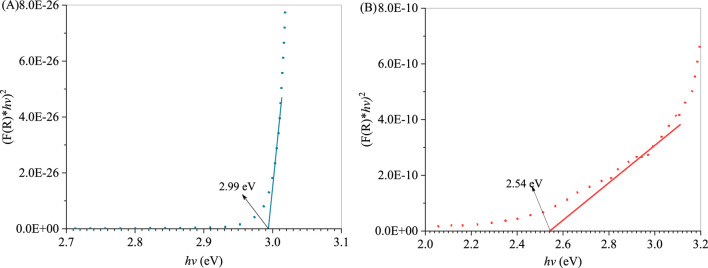
Correlation curves of (*Ahν*)^2^ vs. *hν* of CuWO_4_ **(A)** and La_2_(WO_4_)_3_ **(B)**.

The UV-Vis diffuse reflectance spectra (DRS) of CuWO_4_, La_2_(WO_4_)_3_ and LC-10 were measured using a UV-Vis spectrophotometer in the wavelength range of 200–800 nm, and the results were shown in Figure 6. It could be seen that CuWO_4_ had a strong light absorption capacity in the entire 200–800 nm range, indicating that CuWO_4_ had a good light response ability to both UV and Vis light. La_2_(WO_4_)_3_ had an obvious absorption boundary near 340 nm, which indicated that La_2_(WO_4_)_3_ had a strong absorption capacity for UV light. It could be seen from the DRS of LC-10 that La_2_(WO_4_)_3_/CuWO_4_ composite had a good light response to both UV and Vis light. Based on the above DRS results, the band gap energy (*E*_g_) of CuWO_4_ and La_2_(WO_4_)_3_ could be obtained by the Kubelka-Munk formula (Wang et al., 2021). The correlation curves of (*Ahν*)^2^ vs. *hν* of CuWO_4_ and La_2_(WO_4_)_3_ were shown in Figures 7A, B, the *E*g of CuWO_4_ and La_2_(WO_4_)_3_ were obtained as 2.99 and 2.54 eV, respectively.

### 3.2 Sonodynamic anti-glioma activity of La_2_(WO_4_)_3_/CuWO_4_ composite

**FIGURE 8:**
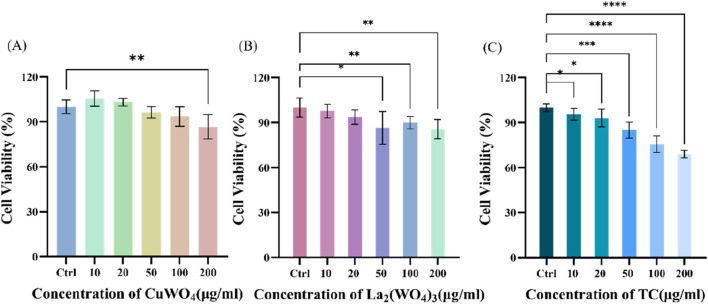
Cytotoxicity of CuWO_4_ **(A)**, La_2_(WO_4_)_3_ **(B)** and LC-10 **(C)** to u251 cells (*p < 0.05,**p < 0.01,***p < 0.001,****p < 0.0001).

First, the cytotoxicity of prepared CuWO_4_, La_2_(WO_4_)_3_ and LC-10 samples to u251 cells was investigated. In the presence of different concentrations (10, 20, 50, 100 and 200 μg/mL) prepared CuWO_4_, La_2_(WO_4_)_3_ and LC-10 samples, the cell viability of u251 cells during logarithmic growth was measured by MTT method. Figure 8A displayed the cytotoxicity test results of CuWO_4_. When CuWO_4_ concentration was 10, 20, 50, 100 and 200 μg/mL, the cell viability was 105.45% ± 5.05%, 103.01% ± 2.57%, 96.31% ± 3.71%, 93.50% ± 6.46% and 86.65% ± 8.08%, respectively. Figure 8B showed the cytotoxicity test results of La_2_(WO_4_)_3_. When the concentration of La_2_(WO_4_)_3_ was 10, 20, 50, 100 and 200 μg/mL, the cell viability was 97.72% ± 4.54%, 93.66% ± 4.80%, 90.84% ± 2.78%, 89.88% ± 4.15% and 85.65% ± 6.49%, respectively. The results showed that CuWO_4_ and La_2_(WO_4_)_3_ did not cause obvious cytotoxicity to u251 cells when the concentrations of CuWO_4_ and La_2_(WO_4_)_3_ were below 200 μg/mL. Figure 8C showed the cytotoxicity test results of LC-10 sample. When the concentration of LC-10 was 10, 20, 50, 100 and 200 μg/mL, the cell viability was 95.17% ± 4.35%, 94.40% ± 6.70%, 84.11% ± 5.31%, 79.26% ± 4.38% and 71.05% ± 6.12%, respectively. These results showed that with the increase of LC-10 concentration, the cell survival rate decreased gradually. When the concentration of LC-10 was higher than 50 μg/mL, it had a certain toxic effect on cells.

**FIGURE 9:**
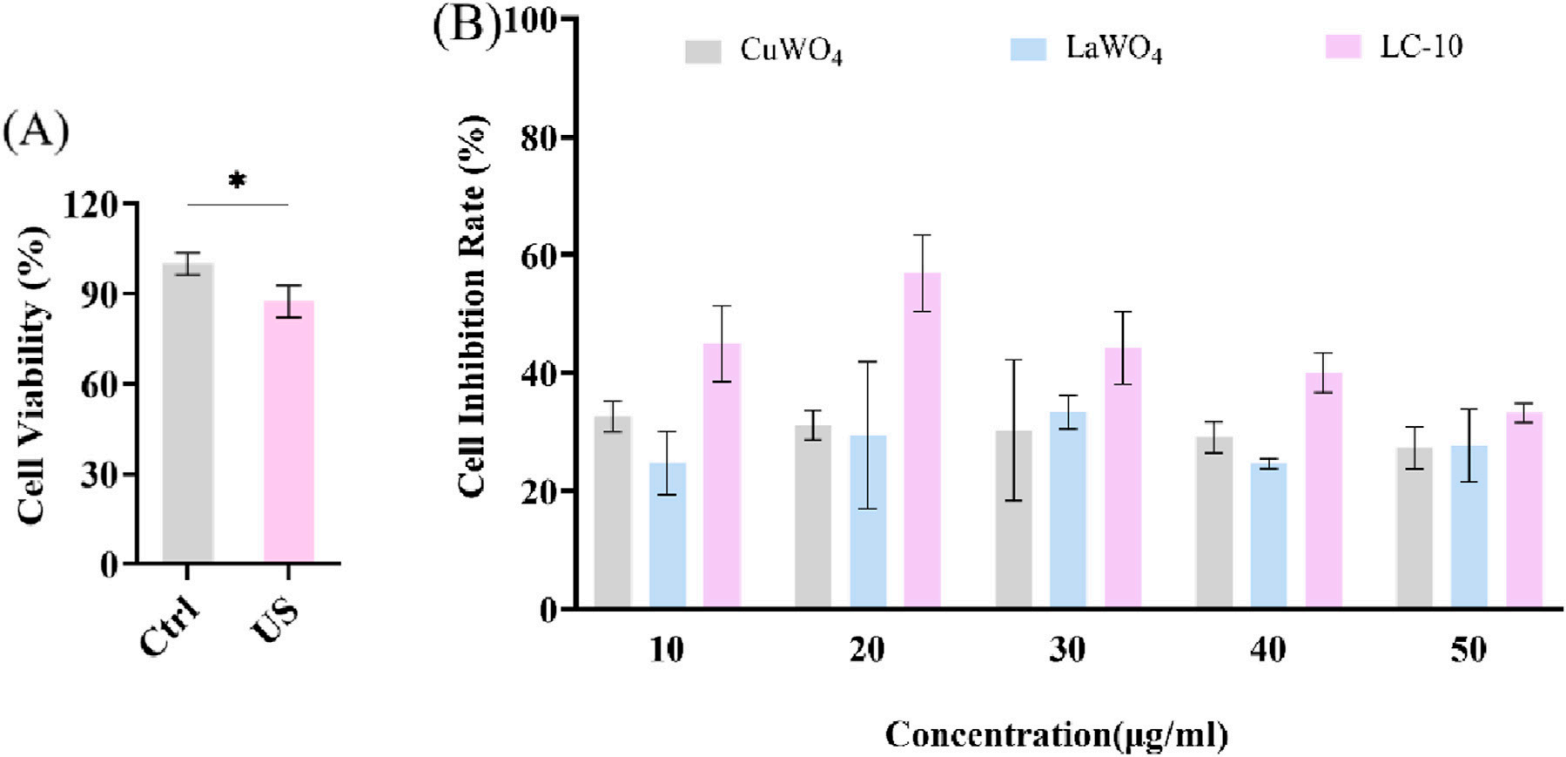
Cell viability of u251 cells under US irradiation **(A)** and cell inhibition rate of u251 cells after SDT in the presence of different concentrations of CuWO_4_, La_2_(WO_4_)_3_ and LC-10 **(B)** (*p < 0.05).

Next, u251 cells were treated differently to evaluate the sonodynamic therapeutic effect of La_2_(WO_4_)_3_/CuWO_4_ composite on tumor cells. It could be seen from Figure 9A that the cell viability decreased slightly under US irradiation alone. This was because the energy generated by the cavitation effect of US could lead to the cracking of water molecules, resulting in a strong oxidizing ·OH. The effects of different concentrations of LC-10 on the cell viability of u251 cells under US irradiation were shown in Supplementary Figures S1–S5. It could be clearly observed that the cell viability of u251 cells treated with SDT in the presence of LC-10 was significantly lower than that treated with LC-10 alone and US alone at any concentration of LC-10. These results suggested that La_2_(WO_4_)_3_/CuWO_4_ composite had excellent sonodynamic antitumor performance. The cell inhibition rate of u251 cells after SDT in the presence of different concentrations of CuWO_4_, La_2_(WO_4_)_3_ and LC-10 were shown in Figure 9B. It could be seen that the cell inhibition rate of u251 cells after SDT in the presence of LC-10 at any concentration was significantly higher than that in the presence of CuWO_4_ and La_2_(WO_4_)_3_ at same concentration. These results indicated that La_2_(WO_4_)_3_/CuWO_4_ composite had higher sonodynamic antitumor performance. In addition, it was also shown that the construction of heterojunction was an effective means to improve the sonodynamic antitumor performance of nano-sonosensitizers.

**FIGURE 10:**
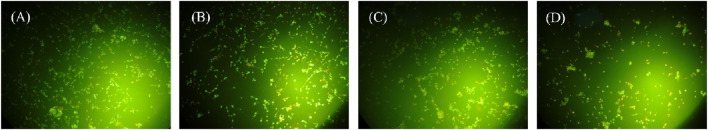
Confocal laser scanning microscopy images of u251 cells stained with AO/EB after various treatments. **(A)** control group, **(B)** US group, **(C)** LC-10 group, and **(D)** LC-10+US group.

Subsequently, sonodynamic-induced cancer cell apoptosis was further evaluated using AO/EB staining. As shown in Figure 10, under the same experimental conditions, cells were divided into control group (A), US group (B), LC-10 group (C), and LC-10+US group (D). In the control group (Figure 10A), the quantity and intensity of green fluorescence were high, while the orange fluorescence was negligible, indicating good growth of cells. As shown in Figures 10B, C, the US group and the LC-10 group showed weak orange fluorescence, and most of the cells showed green fluorescence, indicating that most of the cells had good viability. Figure 10D showed a large amount of orange fluorescence in the LC-10+US group, indicating that LC-10 produced a large number of ROS inducing apoptosis under US irradiation, and the experimental results were consistent with the above MTT results.

### 3.3 Sonodynamic antitumor mechanism of La_2_(WO_4_)_3_/CuWO_4_ composite

**FIGURE 11:**
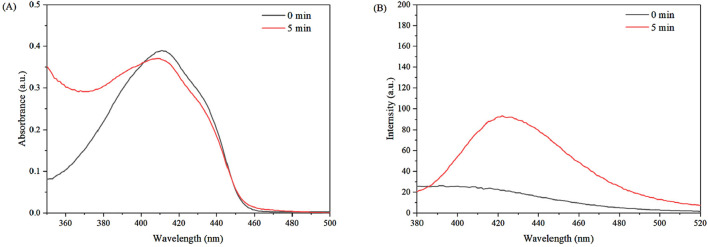
Detection of ^1^O_2_ **(A)** and ·OH **(B)** in the combined use of US and LC-10 system.

It could be clearly seen from the above results that the combined use of US and sonosensiizer LC-10 had a much higher inhibitory effect on the growth of u251 glioma cells than that of US alone and LC-10 alone. The combined use of US and sonosensiizer LC-10 also significantly inhibited the growth of u251 glioma cells compared with the combined effect of US and La_2_(WO_4_)_3_ and the combined effect of US and CuWO_4_, indicating that LC-10 had better sonodynamic antitumor activity. The essence of the good sonodynamic activity of the nano-sonosensitizer was that it could become a ROS generator under US irradiation (Duan et al., 2023). In order to study the ability of La_2_(WO_4_)_3_/CuWO_4_ composite as a sonosensitizer to produce ROS and the corresponding sonodynamic mechanism, specific ROS probes were used to verify the types of ROS produced during SDT process. The ^1^O_2_ yields of La_2_(WO_4_)_3_/CuWO_4_ composite produced under US irradiation were evaluated using DPBF as a probe. It could be seen from Figure 11A, under US irradiation, the absorption peak of DPBF at 410 nm decreased in the presence of LC-10 compared with the control group of water, suggesting the generation of ^1^O_2_ in the system. Similarly, the capture of ⋅OH was tracked by TA. It could be seen from Figure 11B, the combined use of US and LC-10 remarkably increased the fluorescence intensity of the solution around 430 nm. The results showed that La_2_(WO_4_)_3_/CuWO_4_ composite had good ·OH production ability under US irradiation.

**FIGURE 12:**
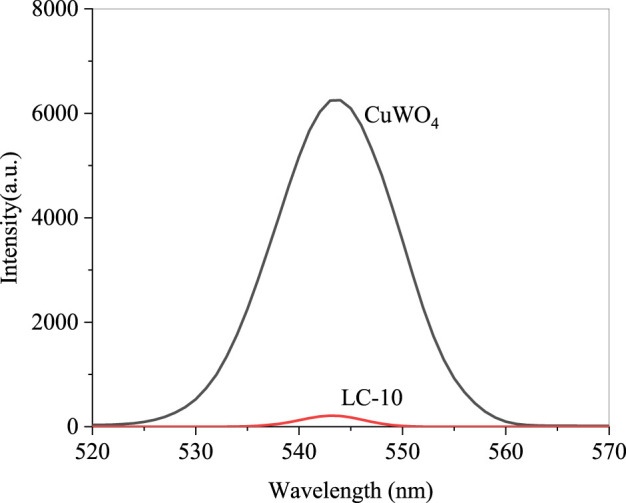
Photoluminescence spectra of CuWO_4_ and LC-10.

The photoluminescence spectra of CuWO_4_ and La_2_(WO_4_)_3_/CuWO_4_ composite LC-10 were measured with a fluorescence spectrophotometer in the wavelength range of 200–600 nm, and the results were shown in Figure 12. It could be seen that under the excitation condition of 269 nm wavelength, CuWO_4_ had an obvious emission peak near 520 nm. Similarly, the maximum emission wavelength of the emission peak of LC-10 was also around 520 nm, but its fluorescence intensity was significantly lower than that of CuWO_4_, indicating that after La_2_(WO_4_)_3_ and CuWO_4_ formed a composite, carrier separation could be effectively realized in the catalyst, and the recombination rate of *e* ^−^-*h* ^+^ pairs was significantly reduced (Liu et al., 2024). The production of ROS of the catalyst under US irradiation was improved significantly, thus displayed excellent sonodynamic antitumor performance.

**FIGURE 13:**
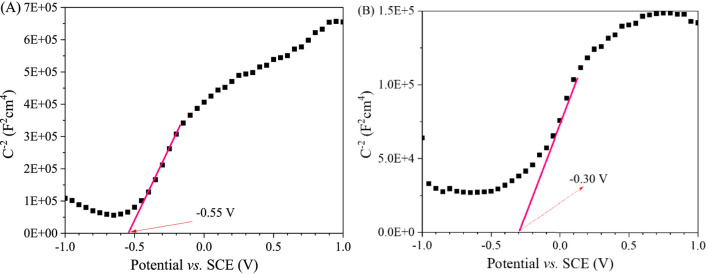
Mott-Schottky plots of La_2_(WO_4_)_3_ **(A)** and CuWO_4_ **(B)**.

**FIGURE 14:**
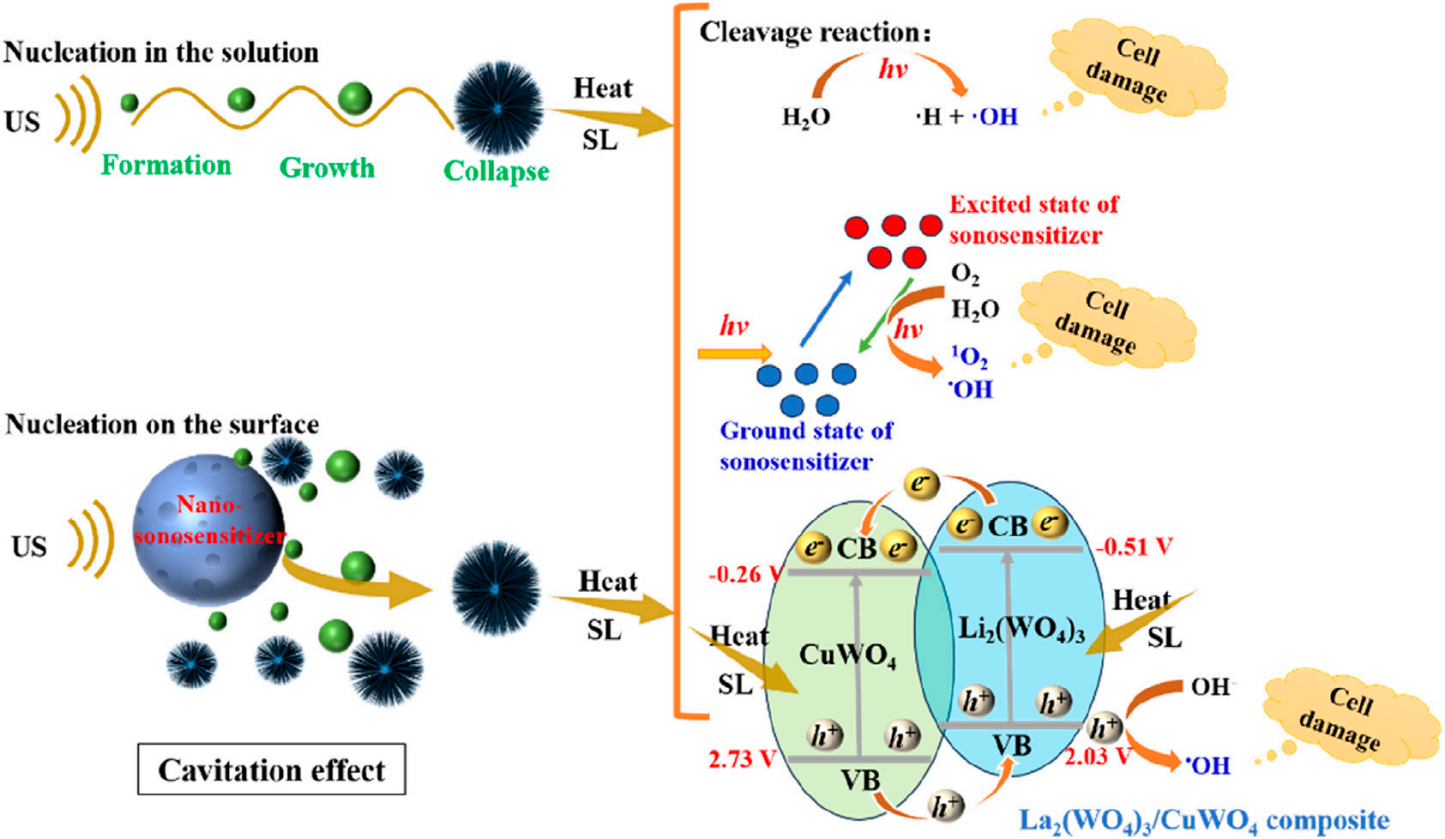
La_2_(WO_4_)_3_/CuWO_4_ composite mediated SDT antitumor mechanism diagram.

The type of semiconductor material could be determined by whether the slope of the tangent in the Mott-Schottky curves was positive or negative. A positive slope indicated an n-type semiconductor and a negative slope indicated a p-type semiconductor (Chang et al., 2023; Chang et al., 2024). As seen in Figure 13, The slopes of tangent lines of the Mott-Schottky curves of La_2_(WO_4_)_3_ and CuWO_4_ were both positive. The results showed that all the prepared La_2_(WO_4_)_3_ and CuWO_4_ samples had n-type semiconductor characteristics. Additionally, the flat band potential (*E*_*f*_) values of the prepared La_2_(WO_4_)_3_ and CuWO_4_ samples could be obtained from the intercept of the tangent with the X-axis (Huang et al., 2024a; Chang et al., 2025). Therefore, the *E*_*f*_ values of La_2_(WO_4_)_3_ and CuWO_4_ samples were determined to be −0.55 V and −0.30 V vs saturated calomel electrode (SCE), respectively. Accordingly, the *E*_*f*_ values of La_2_(WO_4_)_3_ and CuWO_4_ samples could be calculated as −0.31 V and −0.06 V vs standard hydrogen electrode (NHE), considering the correction value between SCE and NHE was 0.24 V (Lu et al., 2025; Huang et al., 2024b). It was known that for most n-type semiconductors, the minimum potential of the conduction band (*E*_CB_) was about 0.1–0.3 V lower than the *E*_*f*_ value, and the middle value of 0.2 V was used here (Huang et al., 2024c; Chen et al., 2024). Therefore, the *E*_CB_ values of La_2_(WO_4_)_3_ and CuWO_4_ samples were calculated as −0.51 V and −0.26 V vs. NHE, respectively. According to the *E*_g_ values of La_2_(WO_4_)_3_ and CuWO_4_ samples were 2.54 and 2.99 eV, respectively, and the relationship between the maximum potential of valence band (*E*_VB_), *E*_CB_ and *E*_g_ (*E*_VB_ = *E*_CB_ + *E*_g_) (Han et al., 2025), *E*_VB_ values of La_2_(WO_4_)_3_ and CuWO_4_ samples relative to NHE could be calculated as 2.03 V and 2.73 V, respectively. Based on the above calculation, the schematic diagram of the band structure corresponding to La_2_(WO_4_)_3_/CuWO_4_ composite was shown in Figure 14. It could be seen that there are obvious differences in the band and energy level structure between La_2_(WO_4_)_3_ and CuWO_4_ in La_2_(WO_4_)_3_/CuWO_4_ composite, and a hypothesis that La_2_(WO_4_)_3_/CuWO_4_ n–n heterojunction formed was proposed.

Combined with the results of the above study and related literature reports, the main mechanism of LC-10-mediated SDT antitumor was proposed (as shown in Figure 14). Firstly, in the process of US irradiation, the tiny bubbles in the liquid were activated by US, resulting in a series of dynamic processes such as expansion, contraction, expansion and collapse, that was, the cavitation effect (Rosenthal et al., 2004; Teng et al., 2024). The cavitation effect (transient cavitation) generated by US could form local hot spots of high temperature and pressure, and its energy could cause water molecules to crack and produced ·OH (Rosenthal et al., 2004), which would cause damage to tumor cells. Secondly, the addition of La_2_(WO_4_)_3_/CuWO_4_ composite nanoparticles could provide more active sites for the nucleation process and the formation of cavitation microbubbles (Qiu et al., 2018), which could generate more active free radicals, and then caused more serious damage to tumor cells. Thirdly, in the process of US irradiation, the cavitation effect of US would lead to sonoluminescence (Xu et al., 2025). The light energy generated by sonoluminescence and the heat energy from local hot spots could activate the sonosensitizer molecule to transition from the ground state to the excited state (Qian et al., 2024). In the process of the sonosensitizer molecule returning from the excited state to the ground state, the released energy was transferred to the water and oxygen molecules in the medium. ROS with strong oxidizing properties such as ^1^O_2_ and ·OH were produced (Qian et al., 2024), thus causing damage to tumor cells. Finally, the La_2_(WO_4_)_3_/CuWO_4_ composite formed by La_2_(WO_4_)_3_ and CuWO_4_ was stimulated by the light generated by sonoluminescence and heat from local hot spots. When the light energy and the heat energy were equal to or exceeded the *E*_g_ of La_2_(WO_4_)_3_ and CuWO_4_ semiconductor, the photothermal generated *e* ^−^ would produce on the VB of La_2_(WO_4_)_3_ and CuWO_4_, and then transferred to the CB, forming *h* ^+^ on the VB of La_2_(WO_4_)_3_ and CuWO_4_, respectively (Xu et al., 2025). Due to obvious differences in the band and energy level structure between La_2_(WO_4_)_3_ and CuWO_4_ in La_2_(WO_4_)_3_/CuWO_4_ composite, the photothermal generated *e* ^−^ on the CB of La_2_(WO_4_)_3_ would transfer to the CB of CuWO_4_, and photothermal generated *h* ^+^ on the VB of CuWO_4_ would transfer to the VB of La_2_(WO_4_)_3_. This could effectively inhibit the photothermal-generated *e* ^−^–*h* ^+^ pairs recombination in La_2_(WO_4_)_3_/CuWO_4_ composite. Because the *E*_VB_ oxidation potential (2.03 V) of La_2_(WO_4_)_3_ was higher than that of ⋅OH/OH^−^ (1.99 V) (He et al., 2025; Sun et al., 2025), *h* ^+^ on VB could oxidize OH^−^ to ⋅OH, leading to oxidative damage of tumor cells. In addition, the CB reduction potential (−0.26 V) of CuWO_4_ was not negative than that of O_2_/⋅O_2_^−^ (−0.33 V) (Wang et al., 2024; Sun et al., 2024), which suggested that the *e* ^−^ on the CB of CuWO_4_ could not reduce O_2_ to superoxide anion radical (⋅O_2_^−^). In summary, the production of ROS such as ^1^O_2_ and ·OH, especially the production of ⋅OH, played an important role in the La_2_(WO_4_)_3_/CuWO_4_ composite mediated SDT antitumor process, which was consistent with the results of previous ROS probe experiments.

## 4 Conclusion

In summary, La_2_(WO_4_)_3_/CuWO_4_ composites were prepared by two-step hydrothermal method and characterized by XRD, SEM, XPS, EDX and DRS. On this basis, using u251 glioma cells as model, the sonodynamic antitumor activity of La_2_(WO_4_)_3_/CuWO_4_ composite LC-10 was investigated by MTT method and AO/EB staining. The results showed that compared with La_2_(WO_4_)_3_ and CuWO_4_, La_2_(WO_4_)_3_/CuWO_4_ composite had better sonodynamic antitumor activity, and LC-10 had good biosafety at concentrations below 50 μg·mL^−1^. After La_2_(WO_4_)_3_ and CuWO_4_ formed La_2_(WO_4_)_3_/CuWO_4_ composite, the photo-thermal generated *e* ^−^ on the CB of La_2_(WO_4_)_3_ would be transferred to the CB of CuWO_4_, and the generated *h* ^+^ on the VB of CuWO_4_ would be transferred to the VB of La_2_(WO_4_)_3_ during SDT. The recombination of *e* ^−^–*h* ^+^ pairs was effectively inhibited, and more strongly oxidizing ROS was produced, inducing apoptosis of u251 glioma cells. In which, ^1^O_2_ and ·OH, especially the production of ⋅OH, played an important role in the La_2_(WO_4_)_3_/CuWO_4_ composite mediated SDT antitumor process. In general, the results of this study would lay a foundation for the design of CuWO_4_ base nano-sonosensitizer and its further clinical application in the study of sonodynamic antitumor. At the same time, it also provided a new strategy for the design and development of novel nano-sonosensitizers with excellent sonodynamic activity.

## Data Availability

The original contributions presented in the study are included in the article/Supplementary Material, further inquiries can be directed to the corresponding author.
