# Supplementary material for: Preparation of La2(WO4)3/CuWO4 composite nanomaterials with enhanced sonodynamic anti-glioma activity
Source: Front Bioeng Biotechnol. 2025 Mar 20;13:1566946. doi: 10.3389/fbioe.2025.1566946 (PMC11965641; doi:10.3389/fbioe.2025.1566946)
Supplement: Supplementary file 1 [file DataSheet1.docx]

**Supplementary Material**

**Preparation of La_2_(WO_4_)_3_/CuWO_4_ composite nanomaterials with enhanced sonodynamic anti-glioma activity**

Fang-Yu Liu^a^, Xin Wang^b^, Ye-Fu Liu^c,*^

*^a^ Liaoning University of Traditional Chinese Medicine, Shenyang 110847, China*

*^b^ Shenyang Key Laboratory for Causes and Drug Discovery of Chronic Diesases, Liaoning University, Shenyang 110036, China*

*^c^ Liaoning Cancer Hospital & Institute, Shenyang, Shenyang 110042, China*

* Corresponding author.

*E-mail address:* [hy8151@163.com](mailto:hy8151@163.com) (Y.-F. Liu)

**1. Instruments**

Phase analysis of CuWO_4_, La_2_(WO_4_)_3_ and La_2_(WO_4_)_3_/CuWO_4_ composite LC-10 was performed using an X-ray diffractometer (XRD) (Bruker D8 Advance, Germany) to determine their crystal structures. The synthesized CuWO_4_, La_2_(WO_4_)_3_ and La_2_(WO_4_)_3_/CuWO_4_ composite LC-10 were analyzed by scanning electron microscopy (SEM) combined with energy dispersive X-ray (EDX) microanalysis (JEOL JSM-IT800, Japan) for elemental analysis and surface morphology characterization. The synthesized CuWO_4_, La_2_(WO_4_)_3_ and La_2_(WO_4_)_3_/CuWO_4_ composite LC-10 were analyzed by X-ray photoelectron spectroscopy (XPS) (Shimadzu Model Axis Supra+, Japan) to determine their chemical valence and elemental composition. The optical properties of the synthesized samples were measured using a UV-visible (UV-vis) spectrophotometer (Shimadzu UV-2550, Japan). The electrochemical characteristics of the catalysts measurements were performed with a standard three-electrode system on an electrochemical workstation (Shanghai Chenhua, CHI660E, China). In MTT assay, the optical density (OD) was measured at 490 nm using a microplate reader instrument (BioTek 800TS, America). In the AO/EB staining experiment, the stained cells were observed and photographed using an inverted fluorescence microscope (Olympus, CKX41, Japan).

**2. Figures**


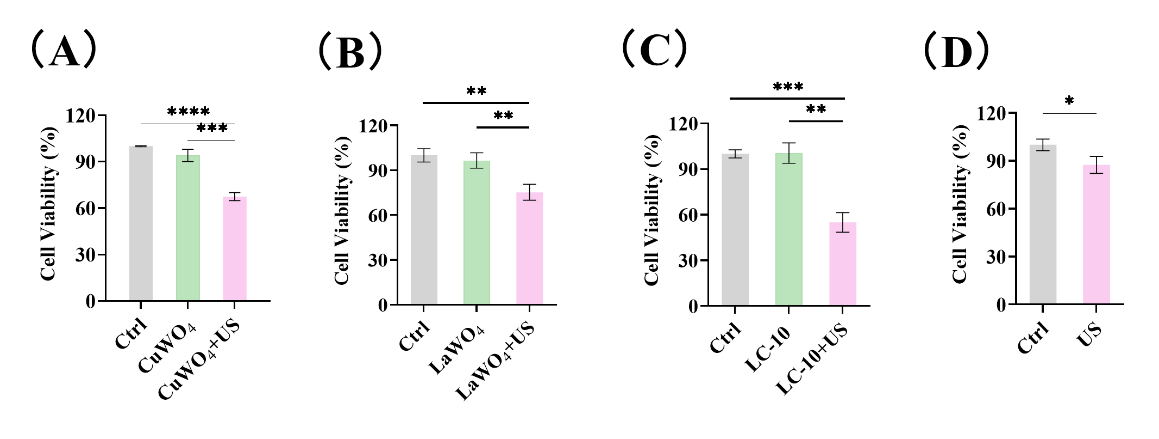


Fig. S1. Cell viability of u251 cells before and after SDT in the presence of 10 μg·mL^-1^ of CuWO_4_, La_2_(WO_4_)_3_ and LC-10 (*p<0.05, **p<0.01, ***p<0.001, ****p<0.0001)


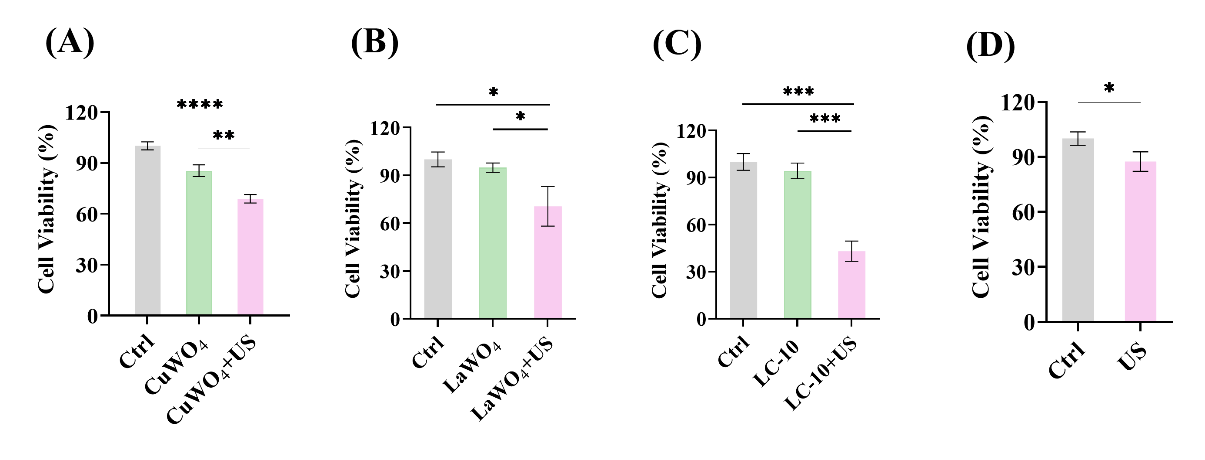


Fig. S2. Cell viability of u251 cells before and after SDT in the presence of 20 μg·mL^-1^ of CuWO_4_, La_2_(WO_4_)_3_ and LC-10 (*p<0.05, **p<0.01, ***p<0.001, ****p<0.0001)


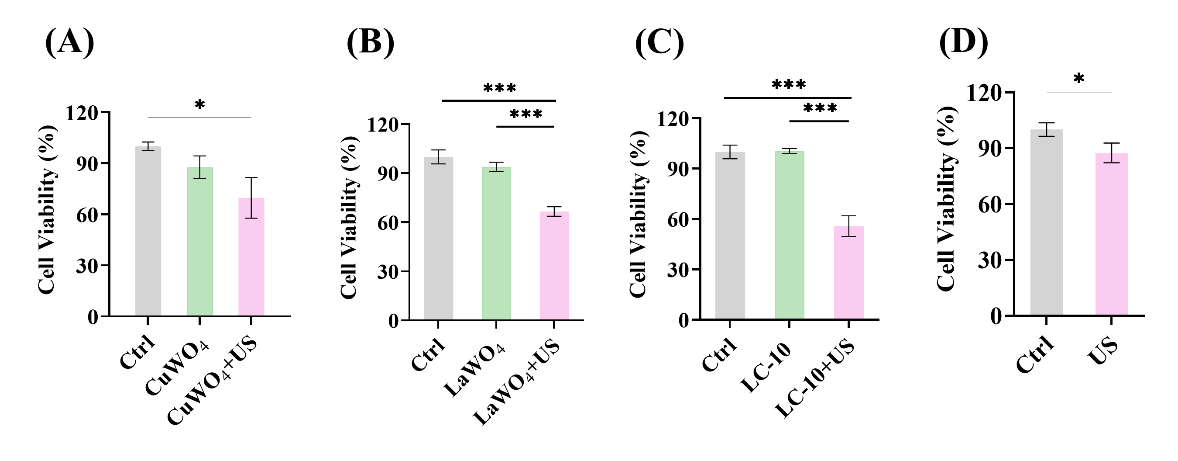


Fig. S3. Cell viability of u251 cells before and after SDT in the presence of 30 μg·mL^-1^ of CuWO_4_, La_2_(WO_4_)_3_ and LC-10 (*p<0.05, **p<0.01, ***p<0.001, ****p<0.0001)


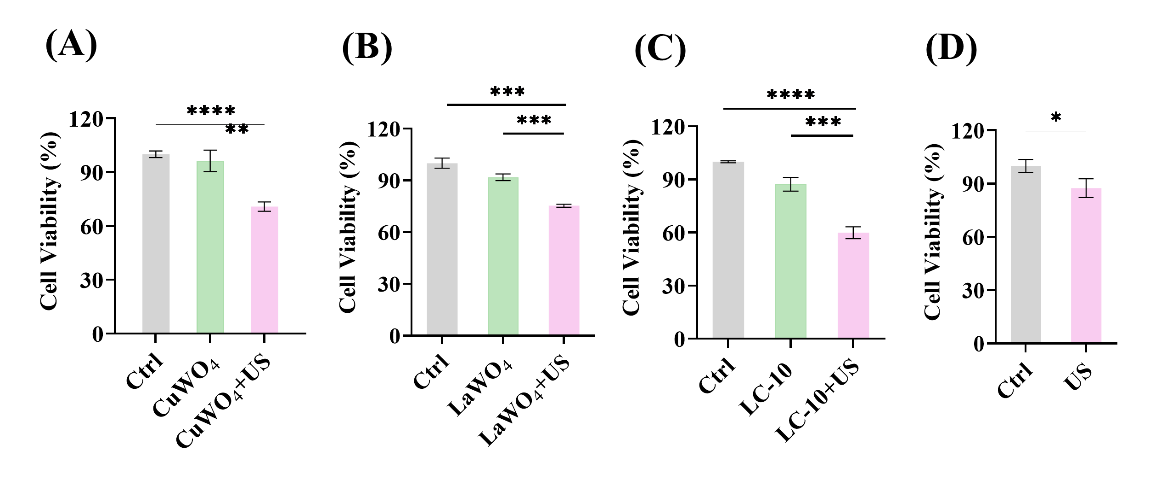


Fig. S4. Cell viability of u251 cells before and after SDT in the presence of 40 μg·mL^-1^ of CuWO_4_, La_2_(WO_4_)_3_ and LC-10 (*p<0.05, **p<0.01, ***p<0.001, ****p<0.0001)


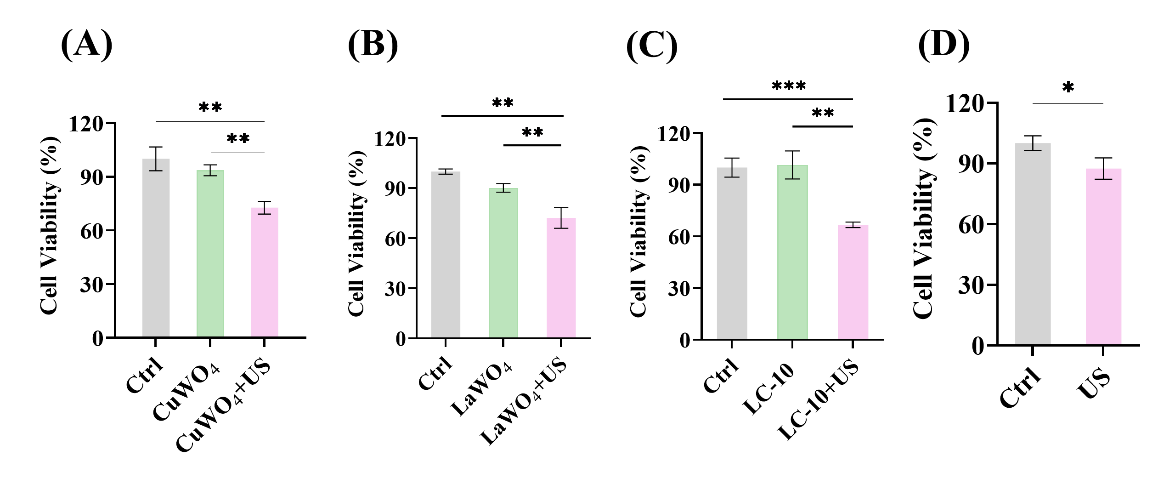


Fig. S5. Cell viability of u251 cells before and after SDT in the presence of 40 μg·mL^-1^ of CuWO_4_, La_2_(WO_4_)_3_ and LC-10 (*p<0.05, **p<0.01, ***p<0.001, ****p<0.0001)
